# In Vitro Cytotoxicity Evaluation of Nanosized Hydroxyapatite and Fluorapatite on Cell Lines and Their Relevance to the Alveolar Augmentation Process

**DOI:** 10.3390/jfb16040125

**Published:** 2025-04-02

**Authors:** Wojciech Zakrzewski, Maria Szymonowicz, Anna Nikodem, Agnieszka Rusak, Zbigniew Rybak, Katarzyna Szyszka, Dorota Diakowska, Benita Wiatrak, Rafal J. Wiglusz, Maciej Dobrzyński

**Affiliations:** 1 Pre-Clinical Research Centre, Wroclaw Medical University, Bujwida 44, 50-345 Wroclaw, Poland; maria.szymonowicz@umw.edu.pl (M.S.); zbigniew.rybak@umw.edu.pl (Z.R.); 2 Division of Biomedical Engineering and Experimental Mechanics, Wroclaw University of Technology, 50-368 Wrocław, Poland; anna.nikodem@pwr.edu.pl; 3 Division of Histology and Embryology, Department of Human Morphology and Embryology, Wroclaw Medical University, 50-367 Wroclaw, Poland; agnieszka.rusak@umw.edu.pl; 4 Institute of Low Temperature and Structure Research, Polish Academy of Sciences, Okolna 2, 50-422 Wroclaw, Poland; katarzyna.szyszka@intibs.pl (K.S.); r.wiglusz@intibs.pl (R.J.W.); 5 Department of Medical Biology, Wroclaw Medical University, 50-368 Wroclaw, Poland; dorota.diakowska@umw.edu.pl; 6 Department of Pharmacology, Wroclaw Medical University, Mikulicza-Radeckiego 2, 50-345 Wroclaw, Poland; benita.wiatrak@umw.edu.pl; 7 Meinig School of Biomedical Engineering, College of Engineering, Cornell University, Ithaca, NY 14853-1801, USA; 8 Department of Pediatric Dentistry and Preclinical Dentistry, Wroclaw Medical University, Krakowska 26, 50-425 Wroclaw, Poland

**Keywords:** hydroxyapatite, fluorapatite, nanosized materials, cytotoxicity, fibroblasts, augmentation, dentistry

## Abstract

**Background/Objectives:** Materials with an apatite structure were investigated in vitro in dental bone augmentation procedures. This scientific study aimed to compare nanosized hydroxyapatite (nHAp) and fluorapatite (nFAp) materials in the form of tablets in in vitro studies, including cytotoxicity assessment and fluoride release. **Methods:** The nHAp and nFAp nanosized materials were obtained using the microwave hydrothermal method. Subsequently, the tablets were prepared from these nanosized powders as further studied materials. Cytotoxicity tests were conducted on Balb/3T3 fibroblast cells and L929 cells. Fluoride ion release was tested at 3, 24, 48, 72, and 168 h periods. **Results:** Both materials presented viability levels above 70%, indicating a lack of cytotoxic potential. The amount of fluoride (F^−^) ions released and accumulated from nFAp was greatly higher than from nHAp. The release of F^−^ ions in both samples was the highest in the first 3 h of exposition. The accumulation of F^−^ ions reached the highest values in the deionized water. The most significant differences in the released or cumulated fluoride ions were observed between deionized water and lower 4.5 pH AS (artificial saliva) samples. **Conclusions:** Both nanosized hydroxyapatite and fluorapatite materials are biocompatible, and their in vitro examination showed promising results for their future in vivo application.

## 1. Introduction

The primary principle of using biomaterials in dentistry is to maintain or enhance oral healthcare [1,2]. The use of biomaterials is continuously evolving in modern dentistry [3]. Due to the increasing complexity of medical procedures and the growing demand for esthetic restorations and procedures, there is an increasing emphasis on the biocompatibility and universality of biomaterials [4,5]. Interdisciplinary approaches adopted by researchers are broadening the horizon of biomaterial applications, maximizing their potential benefits. Biomaterials research is a complicated, multifactorial process that is still rapidly developing. Recent studies confirm a growing interest and need for innovation in dental biomaterials research [2]. Currently, their extensive characteristics allow them to be utilized in a plethora of dental clinical situations, but for the biomaterials to be fully safe for patients, they are subject to strict rules and restrictions [6]. Cytotoxicity evaluation is required to be examined in vitro, prior to in vivo application. During the surgical procedures in dentistry, contact with blood is inevitable [7,8]. Augmentation in dentistry is a procedure aiming to increase the volume of alveolar bone, most commonly necessary before the implant placement if the height and width of the bone are insufficient [9]. After tooth extraction, alveolar ridge loss due to resorption is almost inevitable. Studies confirm that approximately 30% of the alveolar ridge is lost due to the resorption of the alveolar process [10]. After a long period, without tooth structures in the bone, the alveolar process undergoes a gradual, natural degradation process in direct proportion to the time for which the tooth is missing. Many studies have pointed out the efficacy of applying socket-filling biomaterials that can reduce the resorption process [11,12]. In the search for ideal biomaterials, researchers look for one that most effectively restores lost tissues, and apatites, which are naturally present in animal and the human body’s bones, present the highest biocompatible characteristics and have the greatest durability.

Hydroxyapatite (Ca_10_(PO_4_)_6_(OH)_2_, abbreviated as HAp) is the primary inorganic component of bones and teeth [13,14]. Unlike enamel, bone tissue naturally occurs as needles or plates. The calcium-to-phosphorus ratio is 1:67, making it the least soluble form of calcium phosphate found in nature [15,16]. Most importantly, hydroxyapatite can be synthesized artificially. Its derivative, fluorapatite (Ca_10_(PO_4_)_6_F_2_, abbreviated as FAp), has a similar structure, but the presence of fluoride gives the material additional properties [17].

Fluoride reduces acid production and the accumulation of pathogenic bacteria [18,19] in the oral cavity, and plays a crucial role in preventing caries through the long-term, consistent supply of low concentrations of fluoride [20]. The oral cavity is an anatomical structure that naturally creates a plethora of niches for both pathogenic and nonpathogenic microorganisms [21]. Even in immunocompetent patients, pathogenic microorganisms are constantly present on the surfaces of soft and hard tissues, as well as in saliva. Achieving full aseptic conditions during surgical procedures is almost impossible, and due to the high invasiveness of such operations, it is crucial to prevent iatrogenic inflammation. The presence of fluorapatite in bones has been demonstrated to result in a reduction in the critical pH value when compared to that of naturally occurring hydroxyapatite [22,23]. This process plays an important role in enhancing the bone’s resistance to acids produced during bacterial activity. Fluorapatite has been demonstrated to exhibit greater resistance to caries formation when compared to hydroxyapatite.

Nanomedicine is a term relating to the treatment of biological systems with the use of nanosized materials that are engineered constructions in which at least one dimension is equal to or smaller than 100 nm [24,25]. Nanosized materials play an important role in their efficacy during treatment. Such structures show specific chemical and physical properties and interactions with the surrounding environment. It has been confirmed that nanosized materials are much more efficient in filling cavities in conservative dentistry [26]. The high surface-to-volume ratio and molecular interactions allow for an elevated contact surface area with the underlying tissues. This allows them to present elevated chemical reactivity, as well as a high surface-to-mass ratio [27,28]. It is important to underline that nanosized materials meant to be in contact with biological tissues undergo strict rules in order not to provoke toxicity or cause any harm [29]. Thorough physicochemical and cytotoxicity evaluation is needed with respect to the size, surface structure, chemical composition, shape, and solubility [30]. Material evaluation should start from in vitro assays in order to make specific mechanistic and biological pathways that can be tested and isolated under controlled conditions. Additional in vivo studies are crucial as well and should be carried out on animal models [31]. Probable indications of using these selected nanosized apatites in future in vivo bone augmentation procedures include their potential to decrease microbial activity and probable lack of cytotoxicity toward crucial human cells responsible for bone regeneration and remodeling.

It is essential to highlight the unique properties of nanosized materials when compared to conventional materials. Traditional bone graft substitutes, such as microcrystalline hydroxyapatite, β-tricalcium phosphate [32], and bioactive glass [33], have been widely utilized in bone regeneration. However, their efficacy is often limited by factors such as lower bioactivity, reduced surface reactivity, and slower integration with host tissues. In contrast, nanosized materials can offer distinct advantages due to their nanoscale dimensions, which closely mimic the natural mineral composition of bone and teeth. Their high surface area-to-volume ratio enhances cellular adhesion, osteoconductivity, and biointegration, leading to more efficient bone remodeling and regeneration [34,35].

The primary aim of this research was to evaluate the cytotoxicity and fluoride release characteristics of nanosized hydroxyapatite (nHAp) and fluorapatite (nFAp) materials, two promising materials for dental bone augmentation. In vitro cytotoxicity testing was conducted using well-established fibroblast cell lines, Balb/3T3 and L929, which are commonly employed in biomaterial safety assessments. These cell lines serve as a crucial part of cytotoxicity evaluations due to their ability to model key biological responses, including cell proliferation, viability, and morphological changes, in reaction to material exposure. Balb/3T3 cells, derived from mouse fibroblasts, are widely used in toxicological studies due to their high sensitivity to material-induced cytotoxic effects. These cells provide a reliable model to assess the biological compatibility of novel materials, especially in evaluating how the material may affect cell growth and differentiation. The Balb/3T3 cell line is frequently employed to simulate cellular responses to biomaterials in bone tissue applications, allowing for an accurate assessment of whether a material promotes or inhibits the cellular processes crucial for tissue integration and wound healing. These fibroblasts, which play a pivotal role in collagen synthesis and extracellular matrix formation, are instrumental in the remodeling process during bone regeneration and in the initial stages of wound healing. L929 cells, a well-known fibroblast cell line derived from mouse tissue, are equally instrumental in cytotoxicity testing. These cells share similar properties to Balb/3T3 cells and are also utilized to assess cellular response to materials intended for biomedical applications. L929 cells have been extensively studied for their role in evaluating the biocompatibility of a variety of materials, including those used in dental applications. In particular, the cellular responses of L929 fibroblasts are indicative of the safety and potential adverse effects of materials intended for implantation or bone regeneration.

In the context of dental bone augmentation, these two fibroblast cell lines were selected for their critical involvement in the process of tissue integration and bone regeneration. Fibroblasts, as the main contributors to connective tissue formation, are essential for the healing of surgical sites in the alveolar bone and the integration of biomaterials. Therefore, the cytotoxic effects observed in these cell lines are directly relevant to understanding the potential impact of nHAp and nFAp materials on bone healing and regeneration processes. By examining the cytotoxicity and the fluoride release profiles of these materials in vitro, this study provides valuable insights into their safety profile, biocompatibility, and suitability for clinical applications in dental and bone regeneration procedures. The results of this research contribute to a better understanding of the potential benefits and limitations of these materials, guiding future advancements in dental biomaterials research and their use in clinical settings.

To further enhance the significance of selected nanosized materials in clinical dentistry, it is essential to consider future research directions that will contribute to their clinical translation and optimization. While in vitro studies provide valuable preliminary insights into the cytotoxicity and fluoride release characteristics of these materials, in vivo studies are necessary to confirm their biocompatibility, osteoconductivity, and long-term stability in real clinical conditions. Animal models should be employed to assess their integration with alveolar bone and evaluate their impact on bone remodeling processes.

## 2. Materials and Methods

### 2.1. Preparation of nHAp and nFAp Tablets

**Figure 1:**
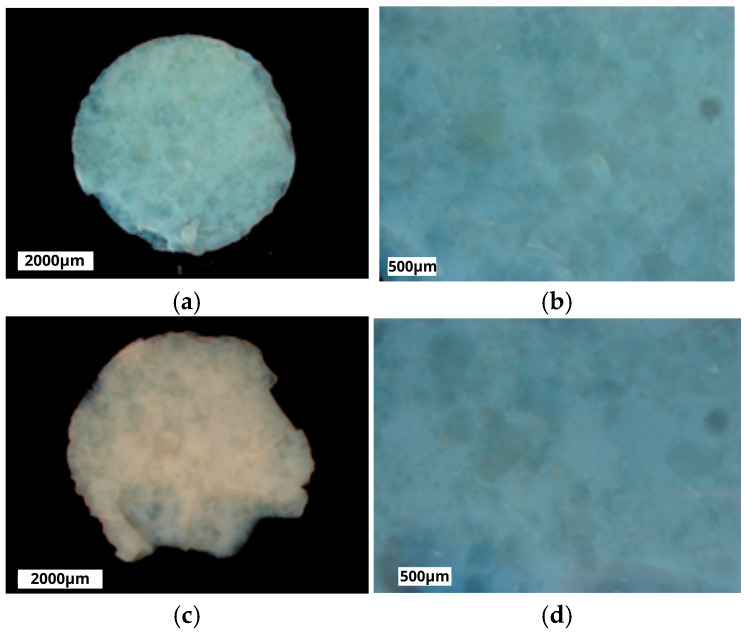
The nFAp (**a**,**b**) and nHAp (**c**,**d**) visualized in microscopic pictures in two different magnitudes.

Both nHAp and nFAp were prepared using the microwave hydrothermal synthesis method. Subsequently, as shown in Figure 1, tablets in the form of disks, 6 mm in diameter and 2 mm in height, were prepared from the previously obtained nHAp and nFAp nanomaterials. Each tablet weighed 0.1 g. Synthesis and tablet preparation were detailed and described in [36].

The physicochemical properties of the obtained nanosized materials were measured using an X-ray powder diffractometer, a scanning electron microscope equipped with an energy-dispersive X-ray spectrometer, and a Fourier transform infrared (FT-IR) spectrometer. The results and detailed discussion are presented in our previous paper [36].

The prepared tablets were sterilized under the following conditions: 134 °C, 2.25 bar gauge pressure, with a holding time of at least 3 min. The tablets then underwent cytotoxicity evaluation, and the release of fluoride over time was measured.

### 2.2. Morphology Characterization

The morphology and elemental composition of the synthesized nanomaterials were analyzed using a field emission scanning electron microscope (FE-SEM, FEI Nova NanoSEM 230; FEI Company as a subsidiary of Thermo Fisher Scientific, Hillsboro, OR, USA) integrated with an energy-dispersive X-ray spectrometer (EDX, EDAX Apollo 40 SDD, EDAX LLC, Pleasanton, CA, USA) operated through Genesis EDAX Microanalysis Software (version 6.0). For the SEM imaging, each sample was dispersed in alcohol, and a droplet of the suspension was deposited onto a carbon stub. The stub was subsequently heated in a vacuum oven at 100 °C for 30 min and allowed to cool to room temperature before analysis. The SEM images were measured at an accelerating voltage of 5.0 kV in beam deceleration mode, which enhances imaging parameters such as the resolution and contrast.

### 2.3. In Vitro Studies

#### 2.3.1. Cell Line

The Balb/3T3 mouse normal fibroblast cell line (clone A31, American Type Culture Collection ATCC^®^ CCL-163TM, Manassas, VA, USA) and L929 mouse normal fibroblast cell line (ATCC) were used, which are both reference models according to standard ISO 10993:5 “Biological evaluation of medical devices—Part 5: Tests for in vitro cytotoxicity” [37,38,39]. The Balb/3T3 cells were cultured in DMEM medium with 4.5 g/L glucose, 25 mM HEPES (Lonza, Basel, Switzerland) supplemented with 10% calf serum, and 1% of L-glutamine solution with penicillin–streptomycin (Sigma-Aldrich, St. Louis, MO, USA). For the L929 cell culture, EMEM medium (Lonza) supplemented with 10% fetal bovine serum (Sigma-Aldrich) and 1% L-glutamine solution with penicillin–streptomycin (Sigma-Aldrich) was used. Cell culturing was conducted at 37 °C in a humidified atmosphere (HERA cell CO_2_ 150i incubator, Thermo Scientific, Waltham, MA, USA) and subculturing every 3 days with TrypLE solution (Thermo Scientific).

#### 2.3.2. Direct Contact

Cytotoxicity evaluation of nHAp and nFAp was performed by the direct contact method, in accordance with ISO 10993:5, using Balb/3T3 cells as a highly sensitive cell model [39]. First, the Balb/3T3 cells were seeded in an amount of 1.5 × 10^5^ cells per well on a 6-well bottom plate (TPP, Trasadingen, Switzerland). After 24 h, a sterile 1 cm diameter test sample from each investigated material was placed on the cells and incubated for the next 24 h. After this, the cell morphology was evaluated under samples, in the nearest of the samples, and in the rest of the wells with an inverted contrast-phase microscope (Olympus, CKX53, Tokyo, Japan). The control cell culture did not come into contact with the investigated materials and was cultured under standard conditions. Changes in the cell morphology and cytotoxicity potential of the materials were assessed.

#### 2.3.3. Indirect Method

The cytotoxicity of the nHAp and nFAp materials was evaluated by extracts obtained from each material, according to standard ISO 10993:12 “Biological evaluation of medical devices Part 12: Sample preparation and reference materials” [40]. In this method, the extracts for the tests for this type of biomaterial were prepared under sterile conditions in the following proportion: 2 g of materials in 10 mL of complete culture medium (with serum—EMEM with 10% fetal bovine serum, L-glutamine, and a mixture of antibiotics (all from Sigma-Aldrich)). This extract was served as a 100% extract and underwent further serial dilutions in complete media (1:1) to 50%, 25%, and 12.5% extracts. High-density polyethylene (HDPE, United States Pharmacopeia (USP) Reference Standard, Sigma-Aldrich) was used as a negative control, as well as sodium lauryl sulfate solution (SLS, Sigma-Aldrich) at concentrations of 0.2, 0.1, and 0.05 mg/mL. The blank was a cell culture without contact with the materials, carried out in a complete culture medium. The solutions prepared in this way were incubated for 24 h. At the same time, L929 mouse fibroblasts—recommended for the indirect contact method [39]—were seeded at a density of 1 × 10 ^4^ cells per well in 96-well plates. Then, after 24 h, the supernatant was removed, and extracts from the tested material at concentrations of 100%, 50%, 25%, and 12.5% and controls were added. After 24 h of incubation, the cell viability was analyzed in MTT assay.

##### MTT Assay

After assessing the morphology of the fibroblast culture, the tested extracts and controls were removed, and 50 µL of the MTT solution with a final concentration of 1 mg/mL, suspended in a medium without adding serum, was added into each well. The plate was incubated at 37 °C for 2 h in a 5% CO_2_ atmosphere. The MTT solution was removed, and 100 µL of isopropanol was added to each well. The plate was then shaken for 30 min until the formazan was completely dissolved. The absorbance of test and control samples was measured on the Varuscan Go spectrophotometer at 570 nm.

The cell viability was calculated according to Formula (1), as follows:

$$V\%=\frac{A_{b}-A_{m}}{A_{s}-A_{m}}\times 100\%$$

where *V*—the cell viability expressed as a percentage, *A_b_*—the average absorbance of the test sample, *A_s_*—the average absorbance of the blank sample, and *A_m_*—the absorbance of the medium. The results are given as a percentage of the control (controls included cells incubated with only a complete medium).

### 2.4. Fluoride Level Release Assessment

The nHAp and nFAp samples were immersed in the meticulously selected media, including artificial saliva (abbr. AS) with a pH scale of 4.5, 7, and 7.5, as well as deionized water, and left in closed containers without mixing. In this scientific work, in order to prepare 1 L of artificial saliva, the following components were used: 0.4 g of NaCl; 0.4 g of KCl; 0.908 g of CaCl·2H_2_O; 0.78 g of NaH_2_PO_4_·2H_2_O; 1 g of urea (CH_4_N_2_O); 0.005 g of Na_2_S·9H_2_O. Deionized water was employed to eliminate potential interactions between fluoride ions and other ions. Due to mimicking the average natural temperature of the human body, all of the tested solutions were incubated at 37 °C. The release of fluoride ions was examined for seven days (168 h) at specific time intervals using an ORION 9609 ion-selective electrode (Thermo Fisher Scientific Co., Waltham, MA, USA) connected to a pH/ion meter, the CPI-551 Elmetron microcomputer. The system was calibrated before each consecutive examination. The measurement was repeated three times, and the mean value was established. Fluoride ion release was tested at the following time intervals: 3, 24, 48, 72, and 168 h. The cumulative values, as well as the levels of F- ions released at specific time intervals, were determined.

### 2.5. Statistical Analysis

The mean and standard deviation (±SD) were used for the presentation of the descriptive data. Student’s *t*-test was used to compare the means between the two study groups. An analysis of variance (ANOVA) for dependent samples or ANOVA for independent groups was performed for multiple comparison procedures, and the post hoc Tukey test was used for intergroup comparisons. To evaluate the association between the time of incubation and the emission of fluoride ions from the nFAp or nHAp tablets, Pearson’s correlation coefficients (r) were calculated. Data for the correlation analysis were logarithmically transformed; *p*-values < 0.05 were assumed as statistically significant. Statistical analysis was performed using MS Excel Professional 2016 (Microsoft Co., Redmond, WA, USA) and Statistica v.13.3 (Tibco Software Inc., Palo Alto, CA, USA).

## 3. Results

### 3.1. Morphology

**Figure 2:**
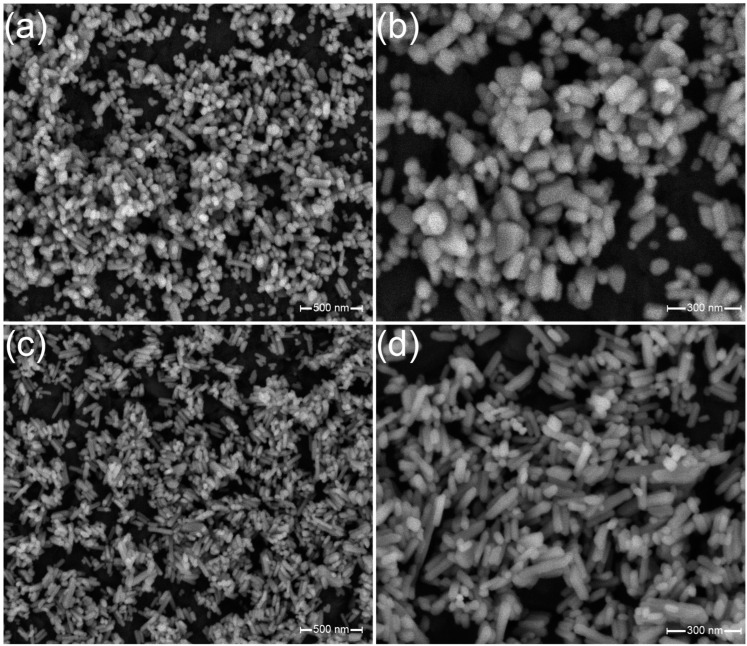
SEM images of the Ca_10_(PO_4_)_6_(OH)_2_ (**a**,**b**) and Ca_10_(PO_4_)_6_F_2_ (**c**,**d**) nanosized powders.

The morphology of the synthesized hydroxyapatite and fluorapatite was imaged using the SEM technique, as shown in Figure 2. The nHAp and nFAp grains have an elongated shape, featuring clear edges and distinct grain boundaries. They are characterized by a rod-like shape, with dimensions in the nanoscale range. The particle size distributions of the examined systems were determined from the SEM images and are presented as histograms in [36]. The average grain size of the nHAp is 56 nm in width and 107 nm in length, whereas the nFAp has an average width of 37 nm and a length of 92 nm. The SEM images clearly confirmed the nanoscale nature of the obtained materials.

### 3.2. Cytotoxicity Evaluation Results

**Figure 3:**
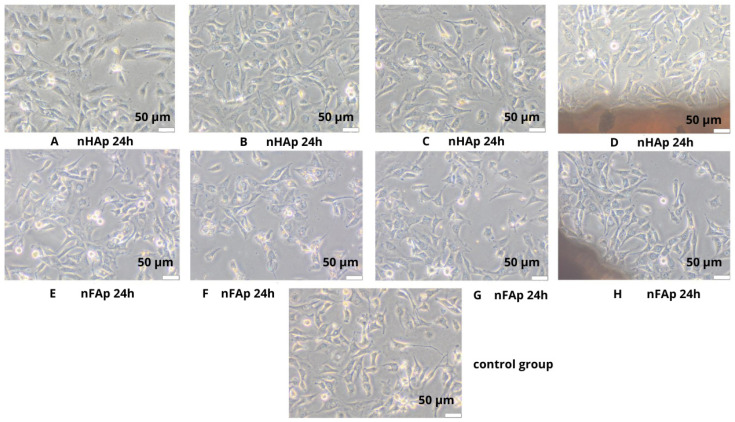
Morphology evaluation of Balb/3T3 cells after 24 h of contact with nHAp and nFAp. (**A**,**E**) cells in the well; (**B**,**F**) near the sample; (**C**,**G**) under the sample; (**D**,**H**) at the edge of the sample. Visualization on inverted contrast-phase microscope. Mag. 100×.

Direct contact of the materials with the Balb/3T3 cells revealed no morphological changes in the cells compared to the control, such as vacuolization, shape change, thinning of the culture, or cell lysis. Thereby, the studied material did not show any cytotoxic potential, as seen in Figure 3, under the samples and also near the material.

**Figure 4:**
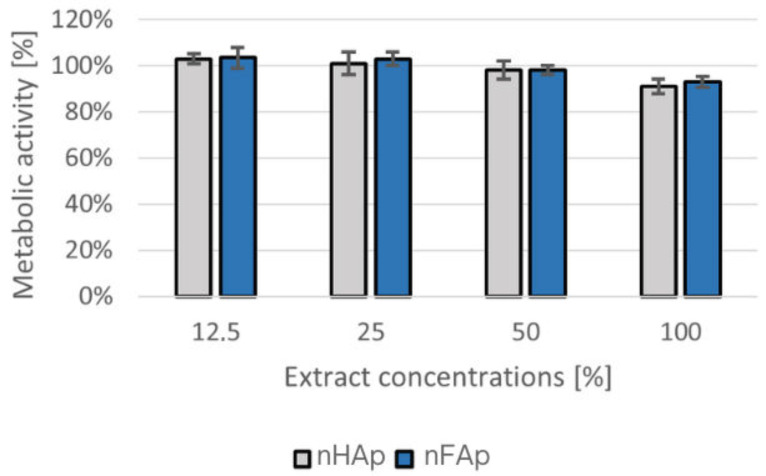
The level of metabolic activity of the L929 cells in contact with the extracts (100%, 50%, 25%, and 12.5%) from the nHAp and nFAp samples after 24 h. Results obtained in the MTT assay with the indirect method of cytotoxicity evaluation.

The effect of exposing L929 cells to the nHAp and nFAp extracts (100%, 50%, 25%, and 12.5%) and control materials for 24 h on the cytotoxicity was determined by the MTT assay, as shown in Figure 4. The control cell group did not have the investigated materials and was cultured under standard conditions. The level of viability of the cells in contact with the extract from the tested material was compared with the level of viability of the cells incubated only in the culture medium. The viability of metabolically active cells in the control group was established at 100%. Following exposure to the tested extracts, cell viability remained above 70% relative to the control. After 24 h of incubation, the viability was measured at 91% for nanosized hydroxyapatite (nHAp) and 93% for fluorapatite (nFAp). The absence of viability reduction below 70% suggests that the tested materials do not exhibit cytotoxic potential. Direct contact of materials with Balb/3T3 cells revealed no morphological changes in the cells compared to the control, such as vacuolization, shape change, thinning of the culture, or cell lysis. Thereby, the studied material did not show any cytotoxic potential.

### 3.3. Statistical Analysis Results

The results of the experiment in Table 1 show the amount of fluoride ions released from the assessed nFAp tablet sample in four distinct environments (deionized H_2_O; AS pH of 4.5; pH of 7.0; pH of 7.5). When all four environments are considered, the highest level of deionized F^−^ ions was observed in the artificial saliva environment with a pH of 7.5, while the lowest values were observed after 186 h of FAp placement in either the artificial saliva or deionized water environments. Among the samples immersed in AS, the highest level of fluoride ions was determined at a pH of 7.5 after the first 3 h (2.806 + 0.531), while the lowest value was observed at the same pH after 168 h. In deionized water, the highest release of fluoride ions occurred after 3 h (1.906 + 0.485), while the lowest release was recorded after 168 h (0.067 + 0.002). Overall, a significant reduction in fluoride ion release was observed over time across all of the tested environments.

Table 2 presents the cumulative release of F^−^ ions from the nFAp material over the same time periods (3, 24, 48, 72, 96, and 168 h) and environments (deionized water, AS pH of 4.5; pH of 7.0; pH of 7.5) as in Table 1. When all four environments are considered, the fastest accumulation level of F^−^ ions was observed in deionized water, with a concentration of 5.718 + 1.457 in the first 3 h and 31.503 + 3.780 after 168 h of exposure. The slowest accumulation of F^−^ ions was observed in the AS solution with a pH of 7.0, with the concentration reaching 0.169 + 0.148 after the first 3 h and 1.680 + 1.075 after 168 h. Among the samples of AS, the fastest accumulation of F- ions was observed at a pH of 7.5 after the first 3 h, reaching 8.418 + 1.594 values at the end of the research (168 h). The slowest accumulation was observed at a pH of 7.0, where the results were 0.169 + 0.148 after the first 3 h and 1.680 + 1.075 after 168 h. A significant accumulation of F^−^ ions was observed in all tested samples, although the highest cumulative values were present in deionized water, or, when counting only the AS solution, at a pH of 7.5.

The results of the experiment, presented in Table 3, show the release of fluoride ions from the assessed nHAp tablet into four distinct environments (deionized H_2_O; AS pH of 4.5; pH of 7.0; pH of 7.5). Among all environments, the highest level of F^−^ ions was observed in deionized water (0.178 + 0.058) after 3 h, while the lowest values, below the detection limit of the electrode, were recorded at most time points in the AS solutions with a pH of 7.0 and a pH of 4.5. In the AS solutions, the highest release of fluoride ions was measured at a pH of 7.5 after 3 h (0.104 + 0.012), while the lowest was observed in almost all hours of the examination of the AS at a pH of 7.0 and the AS at a pH of 4.5. In deionized water, however, the highest level of fluoride ions was determined after 3 h of exposition (0.178 + 0.058). Conversely, the lowest quantity of fluoride ions was released in deionized water after 168 h (0.005 + 0.000). A significant reduction in fluoride ion release was observed over time in all examined nHAp samples. These results clearly show that the highest fluoride ion release occurred in solutions with the highest pH value or in deionized water, while lower pH values, such as 4.5 and 7.0, may have acted as inhibitors of F^−^ ion release.

Table 4 presents the cumulative release of F^−^ ions from the nHAp material over the same time periods (3, 24, 48, 72, 96, and 168 h) and environments (deionized water, AS pH of 4.5; pH of 7.0; pH of 7.5) as in Table 3. When all four environments are considered, the fastest accumulation level of F^−^ ions was observed in deionized water, with a concentration of 0.534 + 0.175 in the first 3 h and 2.797 + 0.874 after 168 h of exposure. The slowest accumulation of F^−^ ions was observed in AS samples with a pH of 7.0, with concentrations so low that they could not be detected by the electrode during the examination. Among the solutions of AS, the fastest accumulation of F^−^ ions was observed at a pH of 7.5 after the first 3 h (0.314 + 0.037), reaching 1.457 + 0.182 values at the end of the study (168 h). A significant accumulation of F^−^ ions was observed in all tested samples, although the highest cumulative values were found in deionized water or, when considering only AS solutions, at a pH of 7.5.

## 4. Discussion

It should be emphasized that each newly developed biomaterial requires multidirectional in vitro evaluation. The present work is a continuation of previous studies focused on apatites’ antimicrobial properties and their influence on the differentiation of fibroblast MC3T3 cells into their successors—osteoblast cells [36]. In contrast to prior studies, the authors performed cytotoxicity evaluation for both nanosized apatites using reference models according to the standard ISO 10993:5, the Balb/3T3 mouse normal fibroblast cell line, and the L929 mouse normal fibroblast cell line. It should be highlighted that the authors used the evaluation of the fluoride release capacity based on the same standards as in the publication by Kosior et al. [41].

In order to assess the clinical utility of future dental materials in an in vitro study, it is essential to replicate the complex conditions present in the oral cavity. The fluoride release levels visibly vary with different pH values, resulting in different results for each medium and its pH level. Saliva plays a pivotal role in maintaining the health and optimal functionality of the oral cavity [42]. It is crucial to emphasize that saliva is a naturally secreted physiological fluid produced by the salivary glands. It performs a multitude of vital functions, including the pre-digestion of food, the remineralization of enamel, and antibacterial activity [43,44,45,46]. Additionally, it provides stability to apatite crystals thanks to the content of calcium, fluoride, and phosphate ions [47]. It is not feasible to develop artificial saliva that will possess the same composition as the natural one, given the multitude of interactions between all salivary components and their variable agents.

The rationale behind the utilization of artificial saliva in this study is to create in vitro conditions that are as stable and as close as possible to the natural conditions present in vivo. The selection of an in vitro model for the research was influenced by several factors. Firstly, diet, age, diseases, or sex can affect the composition of natural saliva [48]. Secondly, artificial saliva was prepared due to its natural counterpart’s challenging nature, resulting in instability outside the oral cavity as well as susceptibility to bacterial colonization [49]. Furthermore, the presence of other ions from human saliva could potentially alter the measurements of F^−^ ions in tested materials. Due to the variability of pH in the oral cavity, which causes the release of different values of fluoride ions [47,50], we elected to utilize three distinct pH variants as well as deionized water in our study in order to assess the impact of pH levels on fluoride release in an environment as close as possible to a natural one. In order to fully comprehend the process of fluoride release, it is essential to recognize that it occurs in two distinct phases. The initial phase, which is characterized by a rapid release of large quantities of fluoride, is known as the “early washout” phase [51]. This is followed by a second phase, during which a steady, low-level release of fluoride occurs.

Research conducted in this work confirms that the highest fluoride ion concentration was recorded within the first three hours of immersion, consistent with the early washout phase. This aligns with the findings of other researchers who have reported an initial burst release followed by a gradual decline [52,53]. The rapid early release can be attributed to the immediate dissolution of surface-available fluoride ions, while the sustained release phase is driven by the slower diffusion of fluoride ions from the inner matrix of the biomaterial. In acidic conditions, the initial washout occurs, yet a greater amount of fluoride is released during the subsequent phase. In neutral solutions, prolonged overall diffusion can be observed following the initial washout [19]. In the experiment, deionized water as well as artificial saliva were employed to assess the fluoride anions released from samples under either neutral or generally controlled conditions. The performed examination shows that the amount of F^−^ ions released in the case of both nHAp and nFAp is reduced in time, with the highest amount present in the first 3 h of observation. The highest release and the fastest accumulation of F^−^ ions was observed in samples of both nHAp and nFAp submerged in deionized water, followed by samples with the highest tested pH of 7.5. With regard to the fluoride ion release from the tested biomaterials, the results indicate that the most efficient fluoride release occurs in deionized water or in AS solution with the highest pH (7.5). The observed highest fluoride ion release occurring in deionized water, followed by artificial saliva at a pH of 7.5, is consistent with the findings reported in previous studies regarding fluoride release levels [54]. Piszko et al. reported that fluoride ion release from a commercial fissure sealant was the most pronounced in artificial saliva at a pH of 7.5 after 168 h, with deionized water also exhibiting significant fluoride release levels [55]. These findings are consistent with the results obtained in this scientific work, and further support the conclusion that fluoride release is enhanced in deionized water and artificial saliva at higher pH levels. Given the confirmed negative impact of nFAp and its released F^−^ ions on bacteria, its use in dental surgical augmentation procedures, bone remodeling, and bone regeneration may facilitate the healing of the treated area.

While this study provides valuable insights into fluoride release from nanosized hydroxyapatite (nHAp) and fluorapatite (nFAp), there are several factors to take into consideration during the interpretation of the results. The use of artificial saliva, although an established method for simulating oral conditions, cannot fully replicate the complexity of natural saliva, which contains additional enzymes and microbial interactions that may influence fluoride release. While the in vitro environment offers a controlled setting, it does not fully account for physiological factors such as salivary flow, temperature fluctuations, and the presence of oral biofilms, all of which can affect fluoride release dynamics in vivo. Additionally, while the pH range that was tested provides important data, it does not fully capture the range of pH fluctuations that naturally occur in the oral cavity due to diet and bacterial activity. This study’s short-term observation period, which primarily focused on the initial fluoride release phase, limits our understanding of the long-term fluoride release and its sustained therapeutic effects. Despite these limitations, the study provides valuable preliminary data that can inform future research. The utilization of deionized water as a testing medium facilitated the observation of fundamental fluoride release behavior, though future studies should consider more complex media to better simulate the ionic composition of natural saliva. The current study’s emphasis on nHAp and nFAp is a solid starting point, and future research should extend it to encompass a more extensive array of fluoride-releasing materials and extended observation periods to more accurately capture long-term fluoride release. The incorporation of mechanical and biological factors, in addition to the exploration of patient-specific variables, will further enhance the clinical relevance and applicability of the findings, ultimately contributing to the advancement of fluoride-releasing materials for dental applications.

The implications of these findings are significant for the development of dental materials designed for prolonged fluoride release. Given the confirmed antibacterial properties of fluoride ions, especially from nanosized hydroxyapatite (nHAp) and fluorapatite (nFAp), these materials hold potential for dental surgical augmentation, bone remodeling, and bone regeneration. The ability of these materials to provide a controlled fluoride release over time could enhance their role in preventing bacterial colonization and promoting tissue healing. Future research should focus on optimizing fluoride-releasing materials for prolonged therapeutic effects, considering variations in pH and salivary composition in different patient populations.

The studies contained in the current article, combined with the results in the previous research [36], provide a comprehensive evaluation of the developed nanosized biomaterials. This gives an opportunity to proceed to the second phase of the research in the future.

## 5. Conclusions

The biomaterials nHAp and nFAp that were examined in this study exhibited considerable promise for utilization in dental bone augmentation procedures. A comprehensive evaluation of their potential for toxicity was conducted, and the results indicated that no cytotoxic effects were observed when exposed to Balb/3T3 and L929 cells, with cell viability maintained above 70%. No observable alterations in cell shape or cell lysis were detected, suggesting that both materials are biocompatible and non-cytotoxic. With regard to fluoride release, nFAp exhibited significantly higher fluoride ion release compared to nHAp, particularly within the first 3 h of exposure. The fluoride release profile was found to be higher in deionized water and solutions with an elevated pH, while fluoride release was reduced in lower-pH environments, such as those associated with bacterial infections or periodontal diseases. These findings indicate the potential of nFAp as a valuable source of fluoride in dental treatments. The findings of this study indicate that both nHAp and nFAp could enhance dental surgical procedures by contributing to bone remodeling and regeneration and by reducing the risk of oral and perioral infections through the controlled release of fluoride. This study demonstrates that these materials are promising candidates for improving clinical dentistry; however, it is important to note that the current in vitro model does not fully replicate clinical conditions. Further research in vivo is required to more fully understand their practical applications in clinical settings.

## Figures and Tables

**Table 1:** Fluoride ion release from nFAp into deionized water and three AS solutions with different pH values. Three samples were prepared for each solution. Descriptive data are presented as the mean + standard deviation (+SD). *: statistically significant.

Time (h)	Deionized H_2_O (µg/mm^2^/h)	AS pH of 4.5 (µg/mm^2^/h)	AS pH of 7.0 (µg/mm^2^/h)	AS pH of 7.5 (µg/mm^2^/h)	*p*-Value (ANOVA for Independent Groups)
3	1.906 ± 0.485	0.356 ± 0.034	0.056 ± 0.049	2.806 ± 0.531	<0.0001 *
24	0.244 ± 0.016	0.051 ± 0.005	0.006 ± 0.005	0.018 ± 0.001	<0.0001 *
48	0.209 ± 0.021	0.048 ± 0.011	0.009 ± 0.001	0.008 ± 0.000	<0.0001 *
72	0.196 ± 0.026	0.027 ± 0.014	0.014 ± 0.008	0.015 ± 0.005	<0.0001 *
96	0.251 ± 0.025	0.016 ± 0.003	0.019 ± 0.014	0.013 ± 0.005	<0.0001 *
168	0.067 ± 0.002	0.005 ± 0.002	0.004 ± 0.003	0.003 ± 0.000	<0.0001 *
Mean + SD	0.479 ± 0.096	0.084 ± 0.012	0.018 ± 0.013	0.477 ± 0.090	-
*p*-value (ANOVA for dependent samples)	<0.0001 *	<0.0001 *	0.096	<0.0001 *	-
post hoc Tukey test	*p* < 0.0001 * for 3 h vs. all time subgroups	*p* < 0.0001 * for 3 h vs. all time subgroups *p* = 0.041 * for 24 h vs. 168 h	-	*p* < 0.0001 * for 3 h vs. all time subgroups	-

**Table 2:** Cumulated release of fluoride ions (µg/mm^2^) from nFAp into deionized water and three AS solutions with different pH values. Three samples were prepared for each solution. Descriptive data were presented as the mean + standard deviation (+SD). *: statistically significant; r: correlation coefficient.

Time (h)	Deionized H_2_O (µg/mm^2^)	AS pH of 4.5 (µg/mm^2^)	AS pH of 7.0 (µg/mm^2^)	AS pH of 7.5 (µg/mm^2^)
3	5.718 ± 1.457	1.068 ± 0.104	0.169 ± 0.148	8.418 ± 1.594
24	10.852 ± 1.794	2.154 ± 0.226	0.311 ± 0.271	8.800 ± 1.613
48	15.881 ± 2.315	3.326 ± 0.513	0.530 ± 0.283	9.012 ± 1.613
72	20.587 ± 2.958	3.977 ± 0.869	0.866 ± 0.497	9.387 ± 1.751
96	26.631 ± 3.577	4.359 ± 0.963	1.341 ± 0.855	9.699 ± 1.880
168	31.503 ± 3.780	4.723 ± 1.123	1.680 ± 1.075	9.936 ± 1.907
Correlation (Pearson test)	r = 0.898 *p* = 0.015 *	r = 0.818 *p* = 0.047 *	r = 0.926 *p* = 0.008 *	r = 0.947 *p* = 0.004 *

**Table 3:** Release of fluoride ions from nHAp into deionized water and three artificial saliva (AS) solutions with different pH values. Three samples were prepared for each solution. Descriptive data were presented as the mean + standard deviation (+SD).

Time (h)	Deionized H_2_O (µg/mm^2^/h)	AS pH of 4.5 (µg/mm^2^/h)	AS pH of 7.0 (µg/mm^2^/h)	AS pH of 7.5 (µg/mm^2^/h)	*p*-Value (ANOVA for Independent Groups)
3	0.178 ± 0.058	0.000 ± 0.000	0.000 ± 0.000	0.104 ± 0.012	<0.0001 *
24	0.009 ± 0.000	0.000 ± 0.000	0.000 ± 0.000	0.010 ± 0.000	<0.0001 *
48	0.015 ± 0.004	0.007 ± 0.012	0.000 ± 0.000	0.000 ± 0.000	0.074
72	0.026 ± 0.012	0.000 ± 0.000	0.000 ± 0.000	0.021 ± 0.006	<0.002 *
96	0.028 ± 0.010	0.000 ± 0.000	0.000 ± 0.000	0.008 ± 0.000	<0.0004 *
168	0.005 ± 0.000	0.000 ± 0.000	0.000 ± 0.000	0.003 ± 0.000	<0.0001 *
Mean + SD	0.043 ± 0.014	0.001 ± 0.002	0.000 ± 0.000	0.024 ± 0.003	-
*p*-value (ANOVA for dependent samples)	<0.0001 *	0.458	-	<0.0001 *	-
post hoc Tukey test	*p* < 0.0001 * for 3 h vs. all time subgroups	-	-	*p* < 0.0001 * for 3 h vs. all time subgroups *p* = 0.006 for 48 h vs. 72 h *p* = 0.018 for 72 h vs. 168 h	-

*: statistically significant; AS: artificial saliva.

**Table 4:** Cumulated release of fluoride ions (µg/mm^2^) from nHAp into H_2_O and three artificial saliva (AS) solutions with different pH values. Three samples were prepared for each solution. Descriptive data were presented as the mean + standard deviation (+SD). *: statistically significant; r: correlation coefficient.

Time (h)	Deionized H_2_O (µg/mm^2^)	AS pH of 4.5 (µg/mm^2^)	AS pH of 7.0 (µg/mm^2^)	AS pH of 7.5 (µg/mm^2^)
3	0.534 ± 0.175	0.000 ± 0.000	0.000 ± 0.000	0.314 ± 0.037
24	0.743 ± 0.181	0.000 ± 0.000	0.000 ± 0.000	0.527 ± 0.037
48	1.105 ± 0.294	1.176 ± 0.306	0.000 ± 0.000	0.527 ± 0.037
72	1.746 ± 0.599	1.176 ± 0.306	0.000 ± 0.000	1.033 ± 0.182
96	2.425 ± 0.846	1.176 ± 0.306	0.000 ± 0.000	1.245 ± 0.182
168	2.797 ± 0.874	1.176 ± 0.306	0.000 ± 0.000	1.457 ± 0.182
Correlation (Pearson test)	r = 0.927 *p* = 0.008 *	r = −0.723 *p* = 0.104	-	r = 0.910 *p* = 0.012 *

## Data Availability

The original contributions presented in the study are included in the article, further inquiries can be directed to the corresponding authors.
